# Supplementary material for: Structural insights into VRC01-class bnAb precursors with diverse light chains elicited in the IAVI G001 human vaccine trial
Source: bioRxiv. 2025 May 27:2025.05.22.655646. Preprint. [Version 1] doi: 10.1101/2025.05.22.655646 (PMC12154976; doi:10.1101/2025.05.22.655646)
Supplement: Supplement 1 [file media-1.pdf]

Supplementary Material

Structural insights into VRC01-class bnAb precursors with diverse light chains elicited in the IAVI G001 human vaccine trial

Xiaohe Lin[a], Christopher A. Cottrell[b,c], Oleksandr Kalyuzhniy[b,c,d], Ryan Tingle[b,c,d], Michael Kubitz[b,c,d], Danny Lu[b,c,d], Meng Yuan[a], William R. Schief[b,c,d,e,1], Ian A. Wilson[a,1]

[a] Department of Integrative Structural and Computational Biology, The Scripps Research Institute, La Jolla, California, USA.
[b] Center for HIV/AIDS Vaccine Development, The Scripps Research Institute, La Jolla, CA 92037, USA.
[c] Department of Immunology and Microbial Science, The Scripps Research Institute, La Jolla, CA 92037, USA.
[d] IAVI Neutralizing Antibody Center, The Scripps Research Institute, La Jolla, CA 92037, USA.
[e] Moderna Inc., Cambridge, MA, 02139, USA.
[1]To whom correspondence may be addressed. Email: wilson@scripps.edu; schief@scripps.edu.

Fig. S1. Conserved CD4bs engagement by bnAbs. Epitope defined by contact residues (as in Fig. 1D) for bnAbs DRVIA57 (PDB: 5CD5), VRC23 (PDB: 4J6R) and N6 (PDB: 5TE6) complexed with HIV gp120-core. See also Fig. 1D for VRC01.

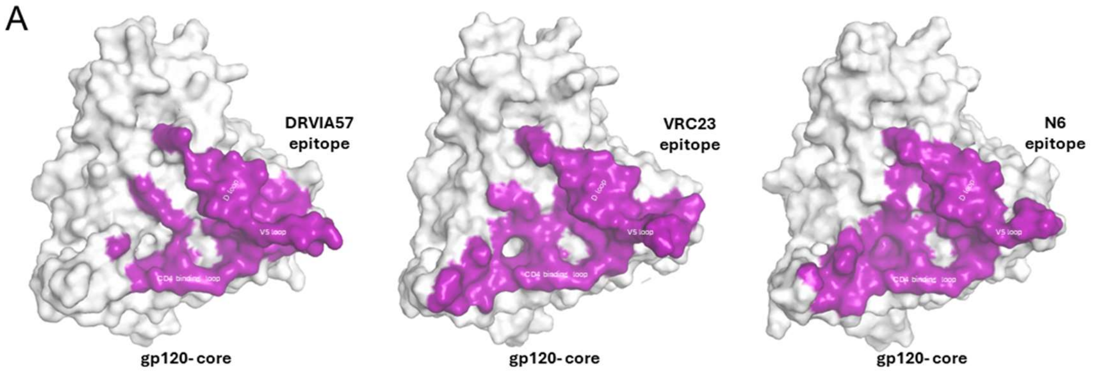

Fig. S2. Conserved heavy-chain interactions across antibody precursors. Zoomed-in view of the IGHV1-2 interaction (as in Fig. 2B) for four G001 antibody precursors (G001-14, G001-58, G001-59 and G001-179). Residues involved in hydrogen bonds are shown in sticks, and conserved hydrogen bonds in both antibody precursor and bnAb VRC01 are indicated by black dashed lines. All other hydrogen bonds are marked by yellow dashed lines.

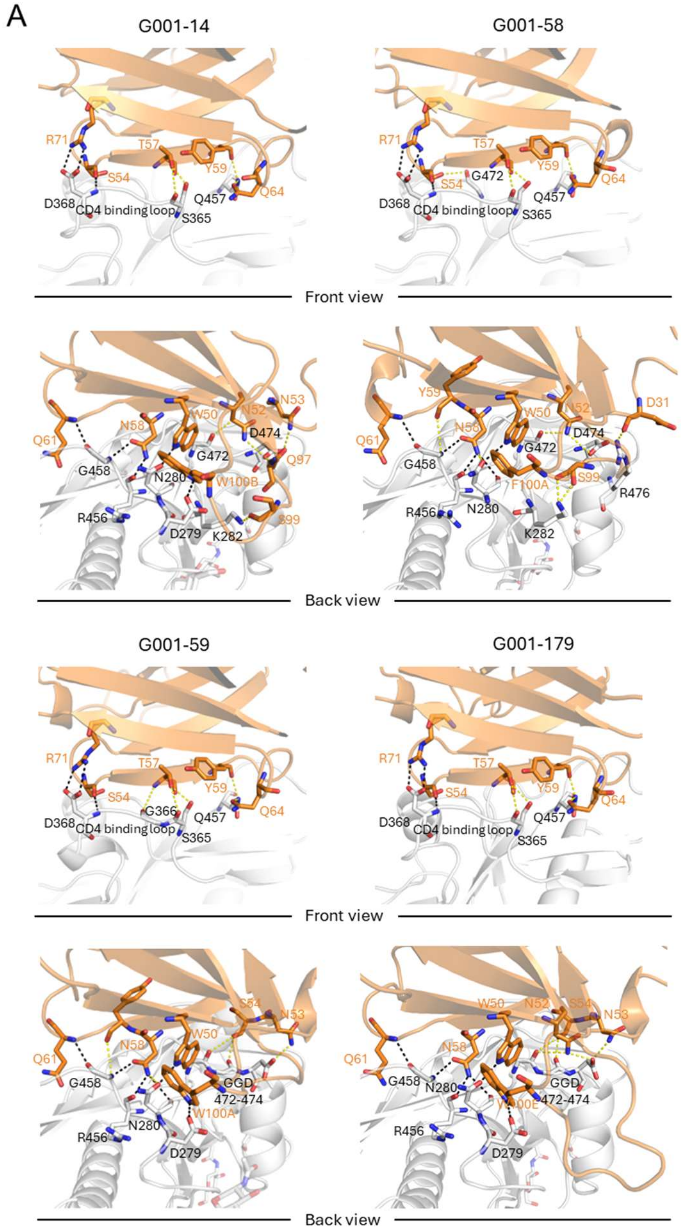

Fig. S3. Light-chain interactions across antibody precursors. Zoomed-in view of light-chain interactions (as in Fig. 3C) for four G001 antibody precursors. (G001-14, G001-58, G001-59 and G001-179). (top: hydrogen bonds; bottom: steric constraints imposed by antigen-proximal regions).

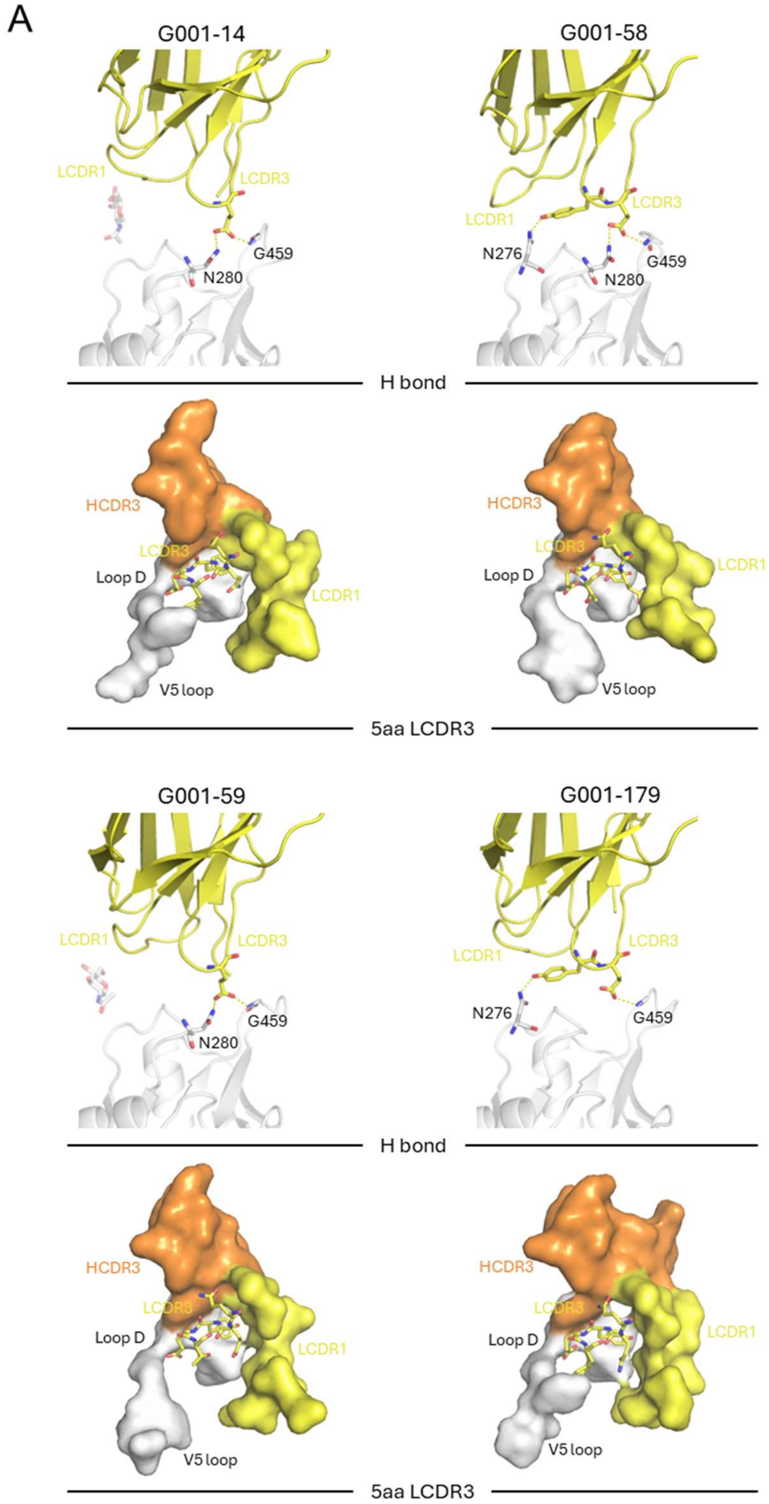

Table S1. Properties of the five VRC01-class bnAb precursors selected for structural analysis.

Antibody name	Timepoint	eOD-GT8 Kon	eOD-GT8 Koff	eOD-GT8 KD (M)	Specimen Type	Heavy V gene	Heavy D gene	Heavy J gene	HCDR3 length	Heavy V gene identity (aa)	Light V gene	Light J gene	HCDR3 length	Light V gene identity (aa)
G001_gp1_V10_VRC01c_2021-0087	week 16	4.20E+05	6.20E-05	1.50E-10	PBMC	IGHV1-2*04	IGHD3-10*01	IGHJ4*02	11	91.84%	IGKV3-20*01	IGKJ4*01	5	96.70%
G001_LD_07A_VRC01c_58	week 9	3.98E+05	1.74E-04	4.37E-10	PB	IGHV1-2*04	IGHD5-12*01	IGHJ4*02	13	93.88%	IGKV1-5*03	IGKJ1*01	5	98.90%
G001_LD_07A_VRC01c_59	week 9	3.94E+05	6.93E-04	1.76E-09	PB	IGHV1-2*04	IGHD6-13*01	IGHJ4*02	13	95.92%	IGKV3-15*01	IGKJ1*01	5	98.90%
G001_gp1_V10_VRC01c_179	week 16	4.50E+05	5.90E-05	1.30E-10	PBMC	IGHV1-2*02	IGHD2-15*01	IGHJ4*02	17	93.81%	IGKV1-33*01	IGKJ3*01	5	98.90%
G001_LD_V05_VRC01c_14	week 3	2.10E+05	7.60E-05	3.70E-10	FNA	IGHV1-2*04	IGHD6-19*01	IGHJ4*02	14	96.94%	IGKV1-33*01	IGKJ4*01	5	98.90%

Table S2. Data collection and refinement statistics.

**Data collection**	eOD-GT8-mingly + G001-0087-Fab	eOD-GT8-mingly + G001-58-Fab	eOD-GT8-mingly-N276 + G001-59-Fab	eOD-GT8-mingly + G001-179-Fab	eOD-GT8-mingly-N276 + G001-14-Fab
Beamline	APS 23-ID-B	SSRL 12-1	SSRL 12-1	SSRL 12-1	SSRL 12-1
Wavelength (Å)	1.0332	0.9795	0.9795	0.9795	0.9795
Space group	I 2 2 2	C 2 2 2_1	I 2 2 2	P 1	C 1 2 1
Unit cell parameters					
a, b, c (Å)	102.5, 136.171, 152.944	110.1, 174.9, 93.6	102.7, 134.0, 154.0	49.2, 65.9, 122.1	261.1, 75.5, 158.1
α, β, γ (°)	90, 90, 90	90.0, 90.0, 90.0	90.0, 90.0, 90.0	89.9, 97.0, 90.0	90.0, 92.4, 90.0
Resolution (Å) [a]	50.00 - 2.60 (2.64 - 2.58)	50.00-2.80 (2.87-2.80)	50.00-2.54 (2.58-2.54)	50.00-1.97 (2.00-1.94)	50.00-2.81 (2.86-2.79)
Unique reflections [a]	33,387 (1,503)	22,255 (1,109)	34,890 (1,696)	96,860 (4,358)	75,138 (3,714)
Redundancy [a]	10.5 (4.0)	12.8 (12.2)	11.4 (6.1)	3.3 (2.8)	6.8 (6.9)
Completeness (%) [a]	99.3 (90.9)	99.1 (99.4)	99.0 (98.3)	89.6 (80.8)	98.4 (99.2)
$<I/\sigma_I>$ [a]	12.3 (1.1)	8.3 (1.0)	14.5 (1.0)	5.8 (2.1)	7.7 (1.3)
R_{sym} [b] (%) [a]	25.9 (115.9)	53.7 (686.4)	16.5 (191.4)	26.6 (139.3)	31.4 (346.6)
R_{pim} [b] (%) [a]	7.6 (49.9)	14.9 (194.8)	4.8 (72.5)	14.3 (78.5)	11.9 (131.3)
$CC_{1/2}$ [c] (%) [a]	98.0 (42.9)	98.7 (41.1)	99.7 (39.3)	95.0 (32.3)	97.8 (31.1)
**Refinement statistics**					
Resolution (Å)	47.74 - 2.58	47.44-2.80	38.50-2.54	26.28-1.94	38.18-2.79
Reflections (work)	33,110	21,156	34,672	96,730	74,974
Reflections (test)	1,631	1,070	1,668	5,082	3,808
R_{cryst} [d] / R_{free} [e] (%)	23.8 / 25.3	21.3/24.9	20.8/23.0	21.3/24.8	21.4/25.4
No. of atoms	4,696	4,541	4,719	10,722	18,474
Antigen	1,288	1,288	1,284	2,576	5,136
Fab	3,228	3,225	3,259	6,448	13,036
Glycan	28	28	42	56	168
Solvent	152	-	134	1,642	134
Average *B*-values ($Å^2$)	48	63	58	23	67
Antigen	56	69	66	22	61
Fab	45	60	54	23	69
Glycan	73	96	94	31	88
Solvent	43	-	50	25	51
Wilson *B*-value ($Å^2$)	44	64	56	18	61
**RMSD from ideal geometry**					
Bond length (Å)	0.003	0.003	0.002	0.007	0.004
Bond angle (°)	0.55	0.50	0.54	0.92	0.71
**Ramachandran statistics (%)**					
Favored	96.4	95.7	97.8	96.6	96.6
Outliers	0.00	0.34	0.17	0.17	0.13
**PDB ID**	9OAO	9OAP	9OAQ	9OAR	9OAS
